# Comparing the Role of the p53 Gene and Telomerase Enzyme in ‘Accelerated Aging Due to Cancer’: A Literature Review

**DOI:** 10.7759/cureus.10794

**Published:** 2020-10-04

**Authors:** Paramvijay Singh Dhalla, Arunima Kaul, Jian Garcia, Anusha Bapatla, Raheela Khalid, Ana S Armenta-Quiroga, Safeera Khan

**Affiliations:** 1 Medicine, California Institute of Behavioral Neurosciences & Psychology, Fairfield, USA; 2 Internal Medicine, California Institute of Behavioral Neurosciences & Psychology, Fairfield, USA

**Keywords:** p53, telomerase, aging, cancer, metformin, statin, p16ink4a, progeria, atherosclerosis, sasp

## Abstract

Aging is defined as progressive physiological alterations in an organism that lead to senescence. In response to stress, when proliferative-competent cells undergo permanent, irreversible growth arrest (like replicative dividing limit, oncogene activation, oxidative stress, or deoxyribonucleic acid (DNA) damage), it is termed as cellular senescence. Biomarkers p53, telomerase, and other inflammatory cytokines have a vital link with senescence, and directed use of these markers might be useful in manipulating cancer and the aging process. We included studies related to topics ' accelerated aging due to cancer', telomerase's relation to Aging and Cancer, p53's relation to Aging and Cancer, Atherosclerosis and Cancer from Search databases like PubMed and Google Scholar. We relied on peer-reviewed articles and included literature from the last 10 years written in the English language. Degenerative diseases in humans are usually linked to atherosclerosis, and atherosclerosis is associated with short leukocyte telomere length. Cancer itself and its treatment are linked with accelerated aging by causing progressive shortening of telomeres during cell replication, resulting in cell death. Gene p53 is known to have a dual effect that works as a tumor suppressor and has pro-aging side effects. In experimental studies, when p53 overcomes multiple regulatory mechanisms controlling its activity, then only the pro-aging side effects of p53 manifested. This might be a potential key for treating cancer without causing the side-effects of aging. In this review, we aim to explain and summarize the interdependent nature of p53, telomeres, and other conventional mechanisms of aging and cancer like inflammation, oxidative stress, uncontrolled proliferation, angiogenesis, micro ribonucleic acids (RNAs), and apoptosis, with a more synergistic approach that can help in developing new therapeutics and play a potential role in shaping modern human lifespan and revolutionize cancer treatment.

## Introduction and background

**Figure 1:**
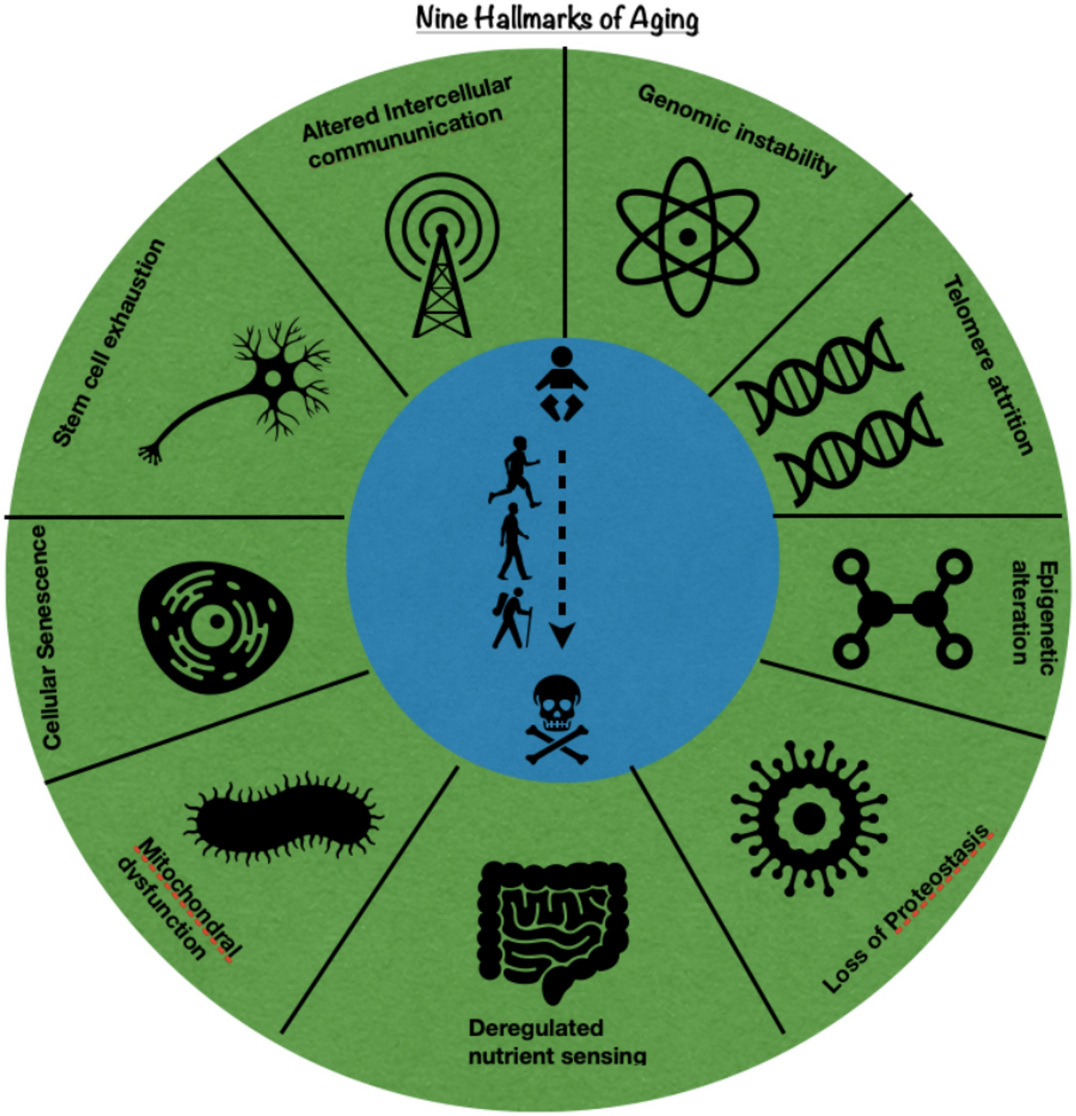
Nine Hallmarks of Aging

Aging is defined as the constant physiological changes in an organism leading to senescence, biological function deterioration, and the capacity to adapt to metabolic stress. 'Senescence' can refer to cellular senescence or whole organism's senescence. Organismal senescence causes an increase in death rates and/or a decrease in fecundity associated with increasing age. In other words, aging applies to degenerative alterations that happen in every organism without any death reference. In contrast, senescence applies to the developmental stage at which 'close to death' manifestations become evident. Manifestations of the aging process include graying of hair, increased susceptibility to infection, higher risk of heatstroke or hypothermia, and musculoskeletal changes. The nine hallmarks of aging are mentioned in Figure 1.

Accelerated aging in cancer

Some studies have investigated accelerated aging in cancer survivors and concluded that accelerated aging manifests as frailty, which is a clinical syndrome in which an individual cannot revert to baseline functional status after a physical insult [1]. Accelerated frailty has been linked to a more striking comorbidity burden in childhood cancer survivors, which was observed in the brains of adult survivors of pediatric lymphoid malignancies and adult breast cancer survivors, than in non-cancer control groups. Compiling evidence supports the hypothesis that cancer itself and its treatment are associated with accelerated aging [1-3]. Aging and cancer have many facets; hence, there are numerous theories, each of which may reveal one or more aging perspectives. One of the notable theories of aging, which we will review in detail, pivots around telomeres, which are repeated segments of deoxyribonucleic acid (DNA) seen at the ends of chromosomes. These decide the maximum life span of a cell because each time a cell divides, many repeats are lost, which shortens telomere, resulting in cell death. Thus, progressive chromosome shortening occurs during cell replication and is observed with aging [4]. Various studies have shown that most degenerative and inflammatory diseases, and many cancers, characterized by oxidative stress, contribute to accelerated telomere shortening [5-6]. As a prerequisite to cellular senescence, a transcriptional regulator called gene p53 causes apoptosis or cell cycle arrest, represses stem and progenitor cell populations' mobilization, resulting in an accelerated aging process. Moreover, p53 also represses unregulated proliferation pathways, leading to senescence-associated secretory phenotype (SASP) and cellular senescence. This generates degenerative and pro-inflammatory tissue milieu resulting in aging process suppression. Henceforth, p53 has the potential to both hinder and expedite cellular aging processes [1,7]. In this review, we will discuss the quantitative expression p16ink4a as a biomarker of aging, used to predict radiation toxicity due to cancer treatment. We aim to explain and summarize the interdependent nature of p53, telomeres, and other conventional mechanisms of aging and cancer with a more collaborative approach, which might aid in understanding these mechanisms from a different perspective.

## Review

Method

We collected free full-text articles as data using PubMed and Google Scholar as our primary databases. We included studies related to the topics 'accelerated aging due to cancer', telomerase's relation to aging and cancer, p53's relation to aging and cancer, atherosclerosis, and cancer. We relied on peer-reviewed articles and included literature from the last 10 years written in the English language. We included all possible types of studies - mixed studies that covered all kinds of ethnicities from around the world. Participants were cancer patients of all ages and gender with signs of accelerated aging like atherosclerosis, gray hair, osteoporosis, and frailty, with biomarkers of aging and cancer like p53 and telomerase enzyme. We excluded non-peer-reviewed articles, literature before 10 years, and articles written in any other language except English. Non-cancer patients with frailty or cancer patients without signs of frailty were also excluded.

Discussion

One of the leading causes of death in both developing and developed countries being cancer. There is a robust biological association between the conventional mechanisms of aging and cancer occurrence. In this review article, we will explain different theories that discuss the evidence on the vital link between aging mechanisms and carcinogenesis. These theories are worth considering, which can help understand the relationship between cancer and the aging process in the general population [8].

Telomerase Enzyme

Telomerase enzyme is a vital enzyme for cell survival that prevents telomere shortening; consequently, cellular senescence is observed after many cell division rounds. By increasing the expression and reactivation of the telomerase complex, cancer cells bypass cellular senescence. This is one of the processes that is required for tumor transformation and progression [9]. Multiple studies have concentrated on identifying strategies or compounds to inhibit telomerase activity in cancer cells with induction of senescence and subsequent loss of telomere integrity [9-11].

Mechanisms of Oxidative Stress Accelerating Telomeric Shortening

**Figure 2:**
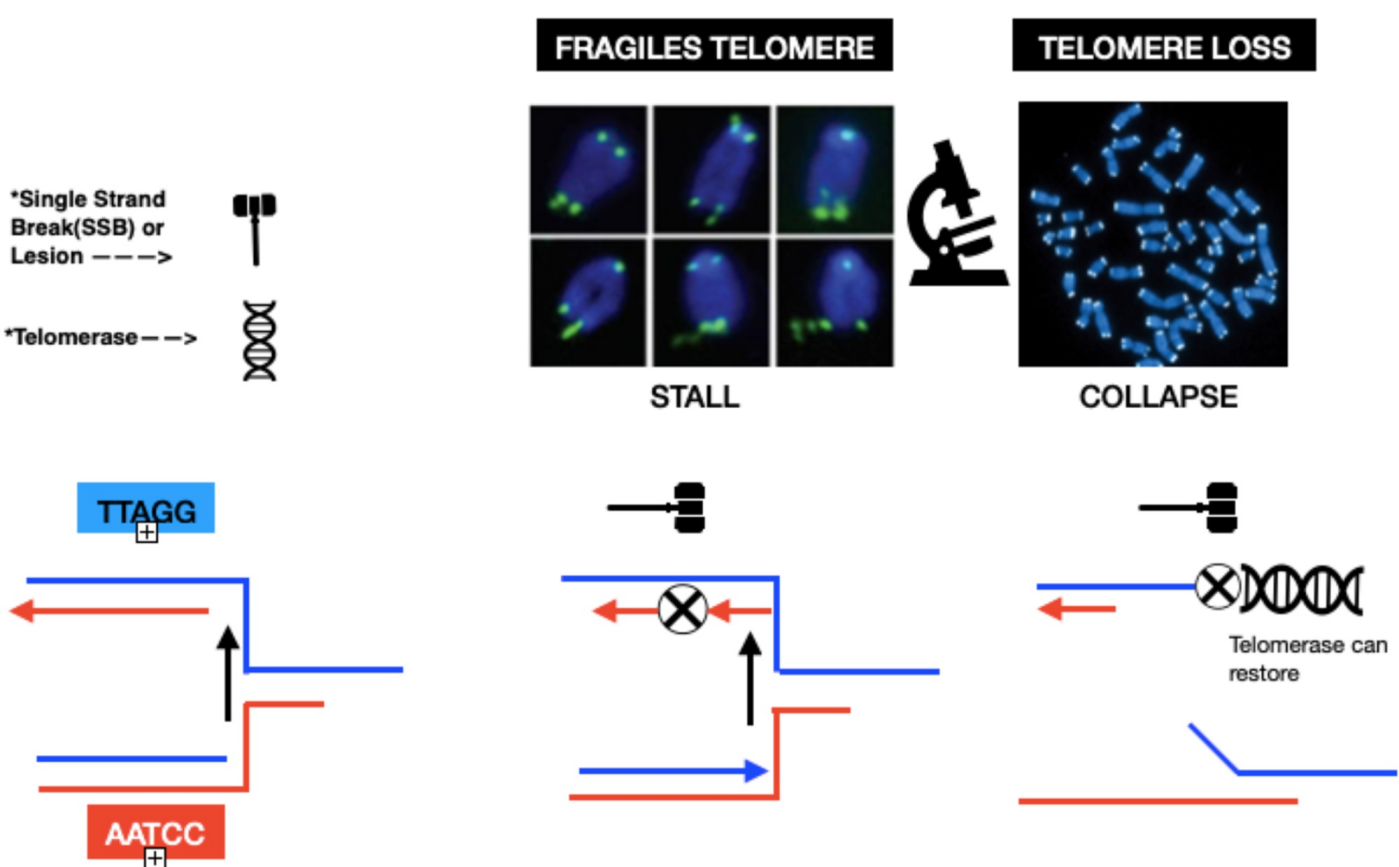
Stalling and Blocking of Telomere's Replication Fork

To demonstrate how oxidative stress accelerates telomere shortening, multiple mechanistic theories have been suggested. One of the theories proposes that oxidative stress triggers cell death or senescence, and to compensate that, the survivors undergo more increased cell divisions, resulting in increased telomere shortening. One of the widely mentioned theories proposes that reactive oxygen species (ROS) causes single-strand breaks (SSB) at telomeres directly or as intermediates during lesion repair, resulting in replication fork collapse and telomere loss (Figure 2). Alternatively, lesions that impede telomere replication can cause an accumulation of unreplicated single-stranded DNA (ssDNA) and manifest as multi-telomeric foci at chromatid ends termed fragile telomeres (Figure 2) [12-14].

Stalling and Blocking of the Telomeres Replication Fork

The schematic exhibits a description of how telomere losses or telomere fragility arises from DNA lesions that collapse or stall replication fork progression, respectively. When the DNA replication fork faces SSBs, it can cause the collapse of the fork, resulting in a double-strand break. Due to uncondensed regions that result from accumulated unreplicated ssDNA, fragile telomeres manifest as multi-telomeric foci at a chromatid end. For detection with a telomeric probe, telomere losses exhibit as chromatid ends lacking sufficient telomeric DNA. By extending a prematurely truncated telomere, telomerase can suppress telomere losses. Hence, telomerase's concept of regeneration can be used for the development of therapeutics (Figure 2).

The Inverse Relation Between Cancer and Atherosclerosis Concerning Telomere Length

**Figure 3:**
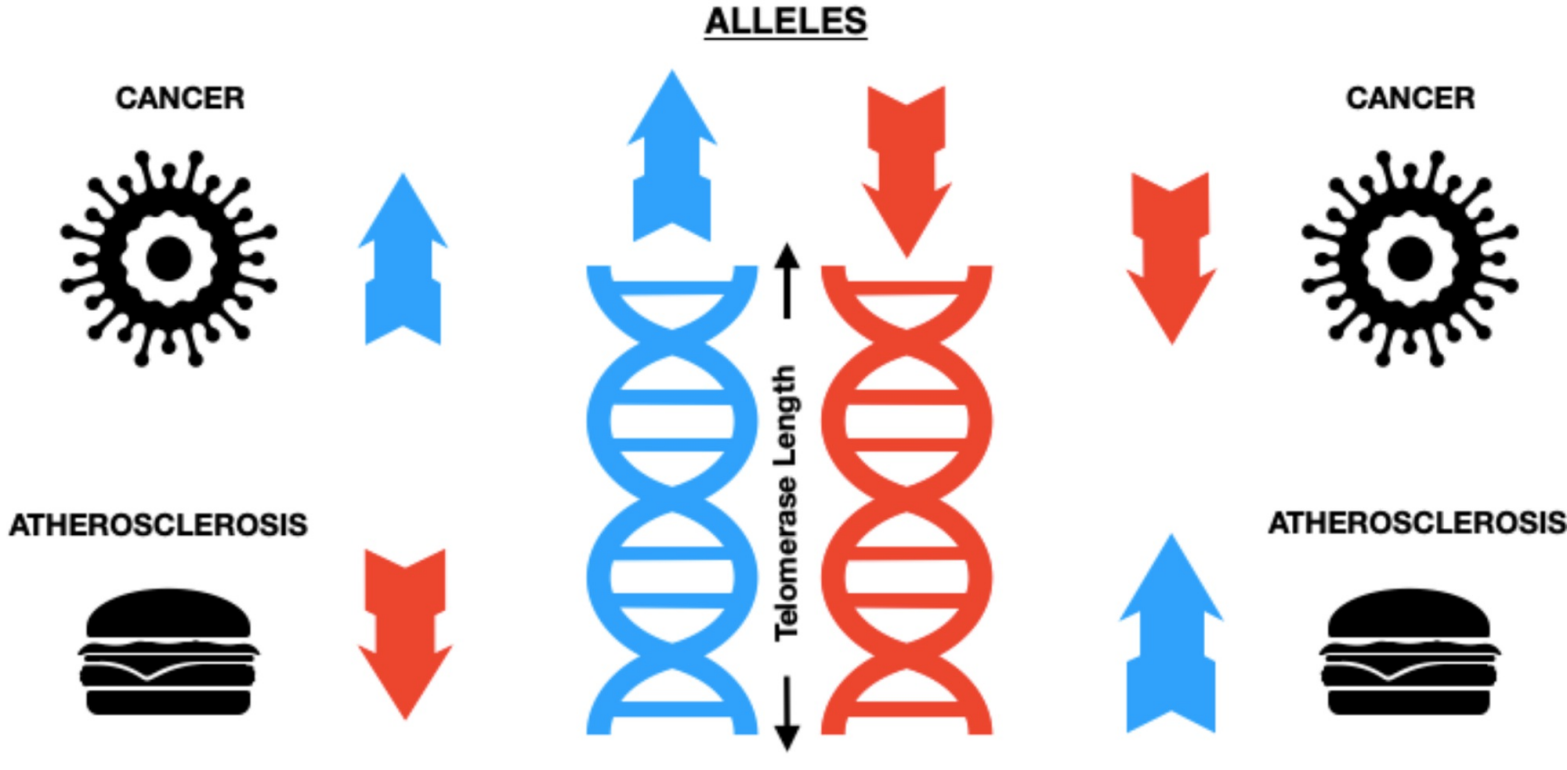
The Inverse Relation Between Cancer and Atherosclerosis Concerning Telomere Length

Degenerative diseases in humans are mostly linked to atherosclerosis, and atherosclerosis is associated with short leukocyte telomere length (LTL) [15-17]. Short telomerase also gives protection against cancer development by diminishing the proliferative activity of stem cells and limiting its regenerative capacity, which gives rise to age-dependent degenerative conditions like atherosclerosis. To create 'genetic risk scores' for cancers and atherosclerosis, which is expressed as coronary heart disease, recent studies have used LTL-GWAS (genome-wide association) study findings [18]. As investigations showing, African Americans have longer telomere length than in European ethnicity individuals; it is interesting to contrast and compare the incidence of notable cancers and atherosclerosis between these two ethnicities. The percentage of cancers like lung (after adjustment for smoking), prostate, pancreas, triple-negative breast cancer, and its incipience at a younger age were greater in African Americans as compared to European ethnicity [19-21]. On a contrary note, African Americans exhibited a lower incidence of atherosclerosis. Hence, emphasizing on the role of telomere length in developing cancers and atherosclerosis more than risk factors itself [22]. In these studies, the LTL-associated alleles (ZNF208, TERC, TERT, OBFC1, ACTP2, RTEL1, and NAF1) seen are a risk sign for melanoma, lung cancer, and coronary heart disease. Therefore, when the alleles' collective effect results in a relatively longer LTL, lung cancer and melanoma risk have risen opposite to the risk of coronary heart disease, which was reduced. Evidencing the fact that increasing telomere length increases cancer risk and decreases atherosclerosis, which is shown in Figure 3. The limitations of these studies are that they don't include other common cancers like breast cancer and colorectal cancer, which might be linked to LTL-associated alleles, and, if not, which other alleles they are linked to should also be researched thoroughly (Figure 3) [23].

p16ink4a Expression Due to Cancer Treatment Influencing Telomere Length

Radiation and chemotherapy can cause progressive and long-term tissue damage by the formation of pro-inflammatory cytokines [24]. The accelerated development of second malignancies and other comorbidities was also seen in survivors of childhood cancer treated with radiations. Chemotherapy has a significant effect on telomere length. Repetitive standard-dose chemotherapy given for solid tumor patients was associated with telomere shortening in hematopoietic stem cells and peripheral blood mononuclear cells [25]. Between the ages of 20 and 80, a more robust marker for predicting molecular aging is the dynamic range of p16ink4a expression, which increases approximately 10-fold. In one study of early-stage breast cancer, in which females treated with adjuvant chemotherapy had their p16ink4a expression estimated in peripheral blood T cells immediately after treatment and was raised by approximately one log2 order of magnitude and continued to be present for one year following treatment [26], which corresponded to approximately a 15-year increase in chronologic age. Undoubtedly, chemotherapy and radiation therapy accelerate aging; hence, patients who are most vulnerable to cancer treatment can be identified by several biomarkers of aging, which are now available and which might minimize treatment-related toxicity by allowing the earlier testing of the interventions. To predict radiation toxicity in individual cases, studies testing how quantitative expression biomarkers like p16ink4a might be utilized for developing possible interventions to ameliorate radiation effects on patients; therefore, they are required to be researched in more detail [27].

p53 Association With Aging

A rare genetic disorder Hutchinson-Gilford Progeria Syndrome (HGPS) is a premature aging disorder that causes de novo point mutation inside exon 11 of the LMNA gene, leading to the accumulation of progerin. HGPS fibroblasts, which are near-senescent, show reduced levels of Δ133p53α and increased levels of p53β. Moreover, due to progerin-induced faulty DNA repair and genomic instability, there is an accumulation of unrepaired DNA and double-strand breaks, which induces cellular senescence [28]. Surprisingly, they found that due to a tumor protection mechanism controlled by BRD4 (Bromodomain-containing protein 4), cells from HGPS patients typically do not develop cancer. Hence, research into the unexplored mechanism of BRD4 and the renewal of Δ133p53α expression can be a crucial link between aging and cancer, which might aid in cancer prevention [29-31].

p53 Isoforms in Cancer

**Figure 4:**
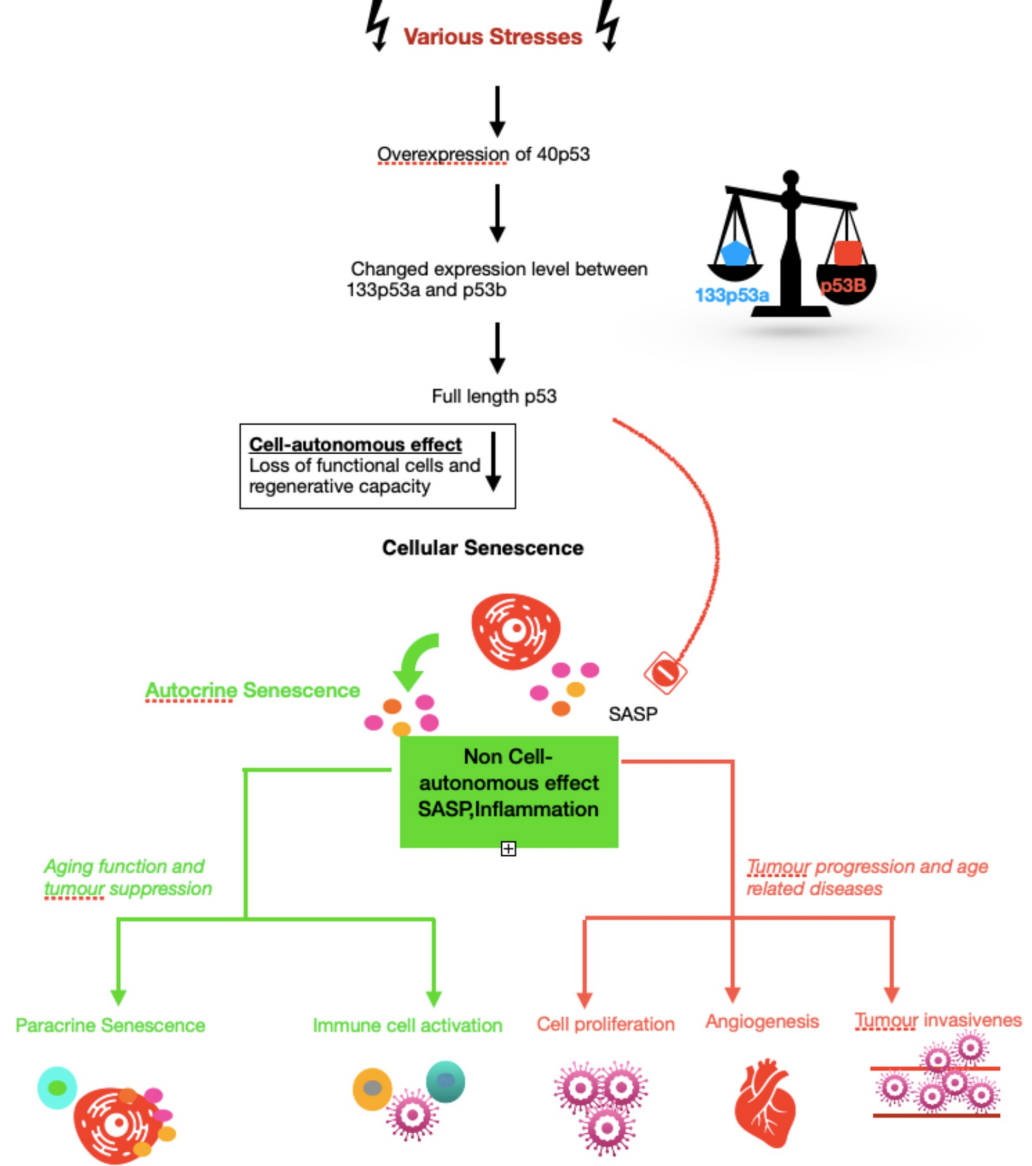
p53 Isoforms in Cancer

The role of p53 in cancer formation is paradoxical. The sudden p53 induction in sarcomas and hepatocellular carcinomas provokes senescence followed by tumor elimination. On the contrary note, cellular senescence has an ability for tumor promotion, which is presumably related to senescence-associated secretory phenotype (SASP) factors. Tumors are arrested at the pre-malignant stage due to cellular senescence. SASP factors are secreted by senescent cells, which promote senescence induction in a paracrine mode (relating to or denoting a hormone that affects only in the vicinity of the gland secreting it) and further reinforces the senescence in an autocrine mode (signifying or relating to a cell-produced substance that affects the cell by which it is secreted) [32]. Through full-length p53 and p53 isoforms, the subsequent model explains the regulation of aging, age-related disorders, and cellular senescence. Depending on the p53 status and cell type, multiple stresses stimulate full-length p53 and change various p53 isoforms expressions. The cell-autonomous effects, like loss of functional cells and regenerative capacity, induce cellular senescence by changing p53 status like decreased D133p53a and increased p53b. The non-cell-autonomous effect, mainly SASP, is also seen in senescent cells. Senescence can be reinforced by autocrine SASP; in turn, the induction of senescence in neighboring cells is achieved by the impact of paracrine SASP and stimulate an immune response, leading to tumor suppression and aging. Concurrently, tumor progression is caused by SASP, which also supports fibrosis, angiogenesis, the proliferation of the cells, and tumor invasiveness. Full-length p53 expressions and various p53 isoforms also regulate this dual outcome through cell-autonomous and non-cell-autonomous functions. Besides, senescence and age-related outcomes require various p53 isoform expression for its occurrence. The full-length p53 desirable outcomes are suppressing pre-malignant tumor cells resulting in tumor suppression, getting rid of damaged cells by stimulating the immune system, and supporting reparative mechanisms like tissue restoration and wound healing [33-35]. However, the deleterious effects of full-length p53 comprise prolonged inflammatory reactions, promoting stem cell-like phenotypes in malignant cells, immune evasion by the tumor, and tumor promotion by inducing angiogenesis [30,32]. The integration of p53 with anti-growth interventions that enhance survival is shown in Figure 4.

The Integration of p53 With Anti-Growth Interventions That Improve Survival

**Figure 5:**
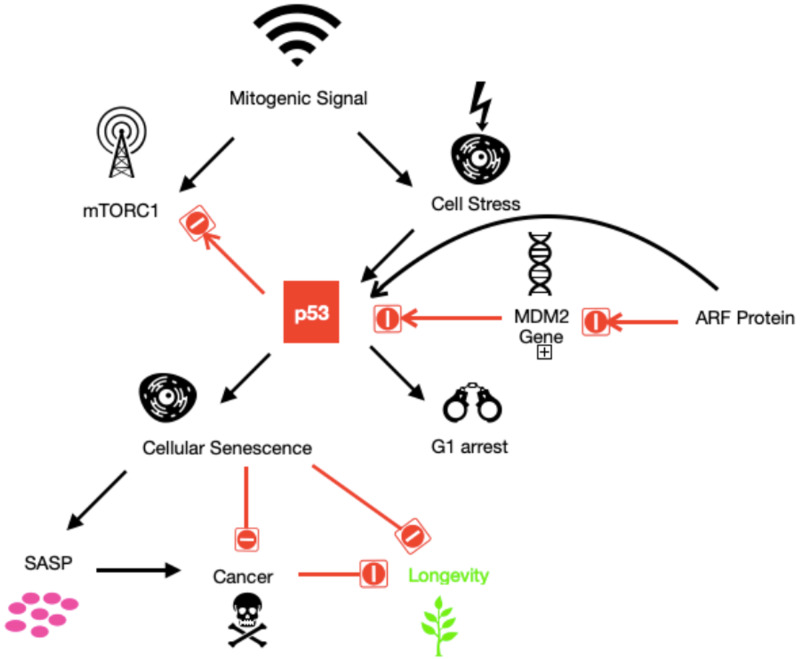
Mitogenic Signals

P53 induces G1 arrest as a part of a stress response, which inhibits mTORC1. G1-arrested cells are enabled by the pro-growth pathway by introducing these mitogenic signals that change into senescent cells and manifest as SASP. Cellular senescence represses the cell from developing into cancer; however, the SASP can trigger cancer development from non-senescent cells by changing the tissue microenvironment. Furthermore, it also decreases longevity and stimulates tissue degeneration. While p53-deletion promotes cancer, simple p53 overexpression should reduce cancer. However, a mice experiment revealed that mice carrying an additional p53 gene within a bacterial artificial chromosome (BAC) exhibited a reduced cancer incidence, with surprisingly no distinctive exaggerated signs of aging. As ARF elevated p53 levels by repressing MDM2 and increased gene dosage of p53 collectively with Arf, it resulted in a decline in cancer rate and improved overall survival. Similarly, increased p53 levels were seen in mice with a hypomorphic MDM2 allele, showing decreased cancer incidence without adverse side effects [7]. These effects are shown in Figure 5.

Therefore, it concludes that enhanced p53-mediated cancer suppression was not toxic to adult mice, and whenever p53 overwhelms various regulatory mechanisms that modulate its activity, only then pro-aging side effects of p53 are exhibited. The limitation of the study mentioned above was that it was not conducted on humans. A broad-based human study on identifying and manipulating these undiscovered regulatory mechanisms should be done, which might be a potential key to treating cancer without the side effects of aging [7]. On the other hand, another study done on mice with a reduced level of mdm2 showed a lack of premature aging phenotype. This suggests that p53 function enhancement is not sufficient to provoke aging. Hence, more study needs to be conducted to understand the underlying physiology to link p53 expression and aging [36]. Various p53 isoforms abnormal expression was also evidenced in breast cancer, ovarian cancer, colon carcinoma, head and neck tumors, glioblastoma, melanoma, renal cell carcinoma, lung cancer, and acute myeloid leukemia, hepatic cholangiocarcinoma, and acute myeloid leukemia [37-39].

Therapeutics Targeting p53 and Myc

A potential approach in pro-senescence therapy is targeting p53 either indirectly or directly and impacting p53 in the senescence process. Hence, trials involving therapeutics activating p53 and/or its pathway are currently under work. One of the strategies tested in tumors that retain wild-type p53 is hindering the MDM2/p53 interaction and improving p53 function. Through the restoration of the p53-mediated tumor suppression pathway, cancer cell growth arrest and apoptosis were induced while generating minimal cytotoxicity and side effects, which has led to the discovery of Nutlin, a precise inhibitor of the p53/MDM2 interaction. Moreover, the effectiveness and side effects of Nutlin-3, such as increases in atherosclerosis, are yet to be established. The signaling pathways involved in the synergistic effects of combining other drugs could also be used to identify additional targets [9]. 5-aminoimidazole-4-carboxamide-1-β-d-ribofuranoside (AICAR) and metformin are compounds that induce adenosine monophosphate-activated protein kinase (AMPK), resulting in the induction of p53. Additionally, metformin inhibits mTORC1 via AMPK, which activates the potential insulin- and IGF-signaling pathways, consequently decreasing the harmful impacts of diabetes mellitus type 2 (including increased risk of cancer). The activation of AMPK and p53 by metformin has shown to inhibit melanoma invasion (but not migration or proliferation), inhibits growth, and improves radiation response for non-small cell lung cancer. Like metformin, AICAR also induces AMPK, resulting in enhancing p53 phosphorylation and promoting p21 to arrest endothelial cells in G0 or G1. Therefore, AMPK-activating agents can potentially induce p53 and decrease mTORC1 to suppress cancer and possibly other diseases [40]. As AMPK-activating agents like metformin have shown to inhibit melanoma invasion, it is a possibility that the p53 gene may have an indirect effect on cancer through the direct effect of IGF-signaling pathways. Hence, this association should be thoroughly researched upon which might help formulate new cancer regimes. Other common causes of aging and cancer and their possible therapeutics are mentioned in Table 1.

**Table 1:** Common Causes of Both Aging and Cancer Formation TNF-α: tumor necrosis factor-alpha; IL: interleukin; TGF-β: transforming growth factor-beta; ROS: reactive oxygen species; CDK: cyclin-dependent kinases; NADH: nicotinamide adenine dinucleotide + hydrogen; NADPH: nicotinamide adenine dinucleotide phosphate; RNS: reactive nitrogen species; VEGF: vascular endothelial growth factor; FGF: fibroblast growth factor; RNA: ribonucleic acid; TNFR1: tumor necrosis factor receptor 1; LDLR: low-density lipoprotein receptor; miRNAs: microRNAs; HIF: hypoxia-inducible factor Source: [45]

Mechanisms	Mediators	Effect	Relation to Ageing & Cancers	Possible Therapeutic interventions
Inflammation	Atherosclerosis antioxidants – Phospholipase A2- Leukotrienes pathway- Cyclooxygenase - TNF-α - Cancer/atherosclerosis IL-6 - TGF-β	A cascade of biochemical events is triggered due to the harmful stimuli that induce the migration of leukocytes from the blood to damaged tissue, resulting in an inflammatory response that causes the growth of the atherosclerotic lesion.	In cancer, macrophages and T cells are the predominant inflammatory cells since they are accountable for the secretion into the microenvironment of massive amounts of inflammatory cytokines, proangiogenic factors, and reactive oxygen species [41].	Interventions involving anti-inflammatory molecule's upregulation like TNF and interleukin-1 receptor antagonist.
Oxidative Stress	ROS's primary endogenous source is the mitochondrial respiratory chain, associated with enzymatic reactions catalyzed by the xanthine oxidase, nitric oxide synthase, and NADH/NADPH oxidase.	Oxidative stress associated with local inflammation, tissue remodeling, endothelial dysfunction, smooth muscle growth, and plaque formation has also been associated with the production of growth factors and mitogens that may stimulate cell proliferation in early atheromatous lesion sites.	As a consequence of the failure of the antioxidant systems, which is responsible for their neutralization, promoting the development of inflammatory processes, there is an imbalance between RNS and ROS contributing to oxidative stress. Which further causes the development of both atherosclerosis and cancer [42].	Interventions involving cell surface receptors, such as CD44 and several integrins that interact with activated macrophages at sites of inflammation, stimulates cell adhesion and migration, consequently, manipulating cancer and the process of metastasis.
Uncontrolled Proliferation	Involves regulatory proteins such as cyclins and CDKs	Macrophages promote the development of atherosclerotic plaque and, in various tissues, stimulate the development of different types of cancer.	Tissue homeostasis is regulated by two of the predominant physiological processes like cell division and programmed cell death, where deregulation of one of them provokes the development of several diseases, including cancer and atherosclerosis [43].	Interventions target deregulation in several control points like the G1-S transition. It is one of the leading causes of accelerated cell growth and accumulated mutations.
Angiogenesis	Activators' growth factor - VEGF. FGF, Cytokines – IL-1, IL-6, IL-8, cathepsin, copper, oncogenes- c-Myc, r Endothelin, erythropoietin, nitric oxide synthase inhibitors cytokines- IL-10, IL- 12, metalloproteinase, inhibitor, zinc, oncogenes - p53, Rb endostatin, interferon-a.	Formation of micro-vessels in an atherosclerotic lesion contributes to the development of plaque, the formation of micro-vessels stimulated by hypoxia, HIF, and ROS have a role in atherogenesis.	The progression of the primary atherosclerotic lesion requires angiogenesis; it is known that the expansion of plaque and its risk complications such as rupture or vascular thrombosis depends on this mechanism. Concerning cancer, tumor vascular development is also essential for proliferation in processes such as metastatic expansion since cancer cells depend on an adequate supply of oxygen and nutrients for this phenomenon to occur, where new blood and lymphatic vessels are formed through angiogenesis and lymphangiogenesis [44].	An example of a medication that blocks VEGF is bevacizumab (Avastin), a monoclonal antibody used in various cancers like colon cancer. It blocks VEGF from binding to the receptors on the cells that line the blood vessels and stop angiogenesis.
MicroRNAs in atherosclerosis and cancer	Both cancer and atherosclerosis proangiogenic - Let7-f, miR-27b, and miR-130a Inhibition of cellular migration, endothelial proliferation, and angiogenesis - miR-221, miR-222.	miRNAs are a group of highly conserved, non-coding small RNAs, which play a crucial role in gene regulation by acting as repressors or activators. Since a single miRNA may have various genes as targets, and several miRNAs may share the same target [45].	In atherosclerosis and cancer, the presence and regulation of several miRNAs have also been associated with the control of cell proliferation, differentiation, and genomic stability, among other functions.	Since miRNAs have been found to regulate atherosclerosis and Cancer development, it becomes crucial to investigate if similar therapeutic strategies involving miRNAs could apply to both diseases.
Apoptosis	The extrinsic or death receptor pathway is triggered by the binding of Fas and TNFR1 to Fas-L. The intrinsic pathway is directed by two groups of molecules, Bcl-2 and Bax.	Apoptosis is characterized by the morphological and molecular changes in cells, including cell shrinking, membrane vesicle formation, and loss of adhesion to neighboring cells.	The process of apoptosis is considered a determining factor in the regression or progression of atherosclerosis by intervening in the stability of the plaque. On the other hand, apoptosis has been recognized as an important player in cancer development since it functions as a molecular tool that cells employ to avoid the proliferation of damaged cells, hence inhibiting tumor growth [46].	Studies investigating LDLR-knockout mice have shown the inactivation of an apoptosis inhibitor expressed by macrophages (Spα/Api6), increasing macrophage apoptosis, and, therefore, inhibiting atherosclerosis.

Therapeutics Targeting Atherosclerosis

Atherosclerosis has been considered a predisposing risk factor for several malignancies, especially of the epithelial type (e.g., prostate, colon, ovary, lung), and a marker of aging. For example, tumors treated with oxazaphosphorines and pyrimidine antagonists decreased the incidence and presence of atherosclerotic lesions. The probable mechanism behind these effects was the changes in the concentration of free cholesterol in the plasma membrane of cells affecting the formation of lipid rafts and caveolae. These can be directly correlated to the function of crucial receptors, such as the epidermal growth factor receptor (EGFR), members of the TNF receptor family, and the tumor necrosis factor-related apoptosis-inducing ligand (TRAIL_ receptor that corresponds well in both cancer development and atherogenesis [47-48]. Combined immunotherapies employing gefitinib or trastuzumab or chemotherapies employing cisplatin or doxorubicin, when coupled with the use of statins, surprisingly resulted in a higher therapeutic response in gastrointestinal and lung cancers. Hence, it was concluded that the synergistic induction of cytotoxicity employing immune- or chemotherapy in conjunction with statins improves the survival rate in patients with ovarian cancer receiving only statins as a way to prevent an adverse cardiovascular event [47,49], as atherosclerosis is the leading risk in aging due to cancer and its treatment which increases morbidity and mortality. A novel therapeutic approach, which should be researched widely, would be the early treatment of atherosclerosis with statins to prevent future risk of cardiovascular events due to cancer and its treatment. As statins are readily available, it would be a game-changing strategy and can be manipulated after more in-depth knowledge of these pathways is available in the future.

## Conclusions

In this article, we have discussed various mechanisms such as telomerase enzyme, p53, and other common mechanisms that indicate that 'accelerated aging' can be caused by cancer and its treatment. The 'double-edged sword' effects of p53 are tumor suppression with pro-aging side effects and tumor promotion with anti-aging effects. However, the pro-aging side effects of p53 usually manifest when p53 overwhelms the many regulatory mechanisms that control its activity. As African American ethnicities have longer telomere length than European ethnicities, African Americans developed more cancer and less atherosclerosis despite having more risk factors for atherosclerosis. This could challenge 'the inverse relation theory between cancer and atherosclerosis concerning telomere length.' Chemotherapy and radiation therapy cause accelerated aging by affecting telomere length; it has been linked with telomere shortening in hematopoietic stem cells. Thus, earlier testing of interventions with quantitative measurement of p16ink4a expression might benefit in minimizing treatment-related toxicity. A new therapeutic strategy researched widely should be the early treatment of atherosclerosis with statins to prevent future cardiovascular events due to cancer and its treatment. This paper's limitation was that there was evidence from several experimental mouse studies, whereas very few human trials have been conducted yet. Finally, we propose platforms for future research on human telomere and p53 genetics due to its potential role in manipulating the human lifespan, which might be a possible solution for treating cancer without the side effects of aging.
